# Supplementary material for: Metformin exposure, maternal PCOS status and fetal venous liver circulation: A randomized, placebo-controlled study
Source: PLoS One. 2022 Jan 28;17(1):e0262987. doi: 10.1371/journal.pone.0262987 (PMC8797196; doi:10.1371/journal.pone.0262987)
Supplement: S1 File — (DOCX) [file pone.0262987.s002.docx]

**The impact of Metformin on the fetal pulmonary and hepatic circulation – a substudy**

This protocol describes a sub-study to be performed at one of the centers (Bergen, Norway) recruiting to the PregMet 2 study. The recruitment, inclusion and exclusion criteria, study medication and pre-scheduled visits are described in the PregMet 2 Study protocol and will not be modified by the following.

*Background*

Metformin is a drug with a complex pharmacological action. Although the primary target organ is the liver, it also affects the myocardial performance, endothelial and renal function. Main metabolic effects include a decreased hepatic glucose production, reduced insulin resistance and inhibition of lipogenesis [[1](#_ENREF_1)]. Metformin also influenced hepatic blood flow in adults with non-alcoholic fatty liver disease by an increase in portal blood flow and decrease in arterial pulsatility [[2](#_ENREF_2)].

Metformin is also showed to influence pulmonary artery endothelial cells (PAEC) from fetal sheep [[3](#_ENREF_3)]. Fetal lambs with in utero pulmonary hypertension (IPH) have PAEC with phenotypical changes that lead to increased reactive oxygen species (ROS) formation and impaired angiogenesis. The association of increased ROS formation with impaired angiogenesis in IPH-PAEC is similar to the findings reported in diabetic endothelial cells [[4](#_ENREF_4)]. In vitro studies have found metformin to act as an AMPK (AMP-activated protein kinase) activator, improving both pulmonary angiogenesis and the bio-availability of nitric oxide (NO) in IPH [[3](#_ENREF_3)].

Metformin passes the placenta barrier and is found in therapeutic concentrations in the fetus [[5](#_ENREF_5)]. In a previous randomised study neonates in the metformin and placebo group did not differ in weight and length at birth, but the metformin group had a larger head circumference [[6](#_ENREF_6)]. A follow up at eight years of age after metformin exposure in utero demonstrated no effects on weight, height and body composition [[7](#_ENREF_7)]. However, Metformin exposure in utero affected the hormone synthesis at birth showing a correlation between maternal and fetal androgen and estrogen levels [[8](#_ENREF_8)] supporting the theory that PCOS, at least in part, may be ‘inherited’ through an altered intrauterine endocrine milieu. In utero Metformin exposure also influenced glucose and lipid metabolism at follow up in 8-year-old children[[7](#_ENREF_7)]. This may be an accidental finding, but could also indicate a permanently altered liver function due to intrauterine metformin exposure.

Women with menstrual irregularity are more prone to suffer from asthma and pulmonary dysfunction [[9-11](#_ENREF_9)]. Menstrual irregularity during fertile age indicates polycystic ovarian syndrome (PCOS), the most common hormonal dysfunction among women [[12](#_ENREF_12)]. PCOS is considered a metabolic disorder showing increased systemic inflammation [[13](#_ENREF_13)]. It is known that PCOS is linked to obesity, type 2 diabetes mellitus, increased risk of cardiovascular diseases, and the condition is also associated with pregnancy disorders like pre-eclampsia and IUGR, pregnancy induced diabetes and preterm birth [[13-15](#_ENREF_13)]. Insulin resistance is an important feature of both PCOS and diabetes, and clearly related to reduced pulmonary function, especially lowered forced vital capacity (FVC) [[16-21](#_ENREF_16)]. Low birth weight is associated with worse adult lung function [[22](#_ENREF_22)]. Intrauterine influences, which retard fetal weight gain may irrecoverably constrain the growth of the airways leading to smaller airways, decreased lung volume, and subsequently to an increased risk of asthma or chronic obstructive pulmonary disease (COPD) throughout postnatal life [[11](#_ENREF_11), [22-26](#_ENREF_22)].

During intrauterine life the liver plays major role in the regulation of fetal growth and metabolism: insulin-like growth factors (IGF 1,2) [[27](#_ENREF_27)] and erythropoietin [[28](#_ENREF_28)] are synthesised in the liver and the organ is a major site of haematopoiesis [[29](#_ENREF_29)]. Experiments indicate a direct relationship between venous liver blood flow, IGF synthesis and subsequent peripheral cell proliferation [[30](#_ENREF_30)]. Furthermore, fetal venous liver flow is modulated by factors like maternal diet, [[31](#_ENREF_31)] body composition [[31](#_ENREF_31)] and weight gain during pregnancy [[32](#_ENREF_32)]. The exposure to an altered intrauterine environment not only affects fetal growth and metabolism: the flow-mediated effect of maternal diet on fat accretion during pregnancy persists into childhood [[33](#_ENREF_33)]. In the light of these findings, also drugs could act as potential modulators of fetal liver flow.

The fetal cardiac output and the proportion distributed to the placenta in normal pregnancies have previously been studied by our group and reference ranges based on standardised techniques have been established [[34](#_ENREF_34), [35](#_ENREF_35)]. Fetal pulmonary artery flow velocity waveforms (acceleration-time/ejection-time ratio) correlate with amniotic fluid biomarkers of fetal lung maturity [[36](#_ENREF_36)]. We previously studied the fetal liver circulation in normal pregnancies and established reference ranges based on standardised techniques [[32](#_ENREF_32), [37-40](#_ENREF_37)]. The present study offers the possibility to elaborate the effect of a potent drug on the fetal circulation.

*Aims*

1. To investigate the effect of Metformin on fetal lung blood flow in the second half of pregnancy.
2. To investigate the effect of Metformin on fetal liver flow in the second half of pregnancy.

*Hypothesis*

1. Intrauterine exposure to Metformin alters fetal lung blood flow.
2. Intrauterine exposure to Metformin alters fetal liver blood flow.

*Methods*

All participants of the PregMet 2 study being recruited at Haukeland University Hospital will be invited to the study. We plan 4 examinations with transabdominal ultrasound at gestational week 19, 28, 32 and 36. Each examination lasts no more than 60 minutes and will include the following measurements:

- Fetal biometry (head circumference, abdominal circumference, femur length)
- Blood flow velocity and pulsatility index of the uterine arteries
- Blood flow velocity and pulsatility index of the umbilical artery (free loop)
- Blood flow velocity and pulsatility index of the middle cerebral artery
- Blood flow velocity and pulsatility index of veins of the ductus venosus
- Blood flow velocity and diameter of the aorta at the level of the aortic valve
- Blood flow velocity and diameter of the pulmonal artery at the level of the pulmonary valve
- Blood flow velocity and diameter of the left and right pulmonary arteries
- Blood flow velocity and diameter of the intraabdominal portion of the umbilical vein (UV).
- Blood flow velocity in the left portal vein
- (Diameter of the ductus venosus (DV))
- (Blood flow velocity and diameter of the main portal stem (PV))
- (Blood flow velocity and pulsatility index of the hepatic artery)

This is also a priority list for the different measurements. At the examination in weeks 36, it is important to complete all measurements stated in the list.

Volume blood flow in the cardiac outlets will be calculated as Q=π(D/2)^2^TAMXV to make the combined cardiac output (COR + COL = CCO). Arterial blood flow to the lungs (Q _lungs_) will be calculated as Q _lungs_ = Q _right_ + Q _left_, where Q _right_ is the volume flow in the right pulmonary artery, and Q _left_ the volume flow in the left pulmonary artery.

Volume blood flow in the umbilical vein (UV) will be calculated as Q=π(D/2)^2^*h*TAMXV (*h*: velocity profile h=0.5 for UV [[41](#_ENREF_41), [42](#_ENREF_42)]. The total venous blood supply of the liver (Q _liver_) will be calculated as: Q _liver_= (Q _UV_ - Q _DV_ ) + Q _PV_.

*Statistical analysis*

The measured values of the flow velocities, flow indices and volume flow will be transformed into z-scores. The metformin and placebo groups will be compared by t-test.

*Power analysis*

In fetuses with growth disturbances a sample size of 30 participants was sufficient to demonstrate differences in blood flow parameters compared to the reference population [[43](#_ENREF_43), [44](#_ENREF_44)]. The effect of metformin may be subtler, and therefore require a larger sample size. We anticipate that the planned number of participants to be recruited in Bergen (n=100) will be sufficient for the study.

*Safety*

There is no scientific evidence that diagnostic ultrasound causes harm to the fetus [[45](#_ENREF_45)]. The examinations will be performed according to international safety guidelines [[46](#_ENREF_46)].

*Ethics*

The measurement results will in general not have any impact on the treatment during pregnancy and delivery. If, however, the ultrasound examination during the study raises the suspicion of fetal compromise of any type, the participant will be referred to the obstetrical or fetal medicine unit for further care.

**References**

1. Viollet, B., Guigas, B., Sanz Garcia, N., Leclerc, J., Foretz, M., and Andreelli, F. Cellular and molecular mechanisms of metformin: an overview*.* Clin Sci (Lond), 2012. 122(6): p. 253-70.

2. Magalotti, D., Marchesini, G., Ramilli, S., Berzigotti, A., Bianchi, G., and Zoli, M. Splanchnic haemodynamics in non-alcoholic fatty liver disease: effect of a dietary/pharmacological treatment. A pilot study*.* Dig Liver Dis, 2004. 36(6): p. 406-11.

3. Teng, R.J., Du, J., Afolayan, A.J., Eis, A., Shi, Y., and Konduri, G.G. AMP Kinase Activation Improves Angiogenesis in Pulmonary Artery Endothelial Cells with In Utero Pulmonary Hypertension*.* Am J Physiol Lung Cell Mol Physiol, 2012.

4. Giacco, F. and Brownlee, M. Oxidative stress and diabetic complications*.* Circ Res, 2010. 107(9): p. 1058-70.

5. Vanky, E., Zahlsen, K., Spigset, O., and Carlsen, S.M. Placental passage of metformin in women with polycystic ovary syndrome*.* Fertil Steril, 2005. 83(5): p. 1575-8.

6. Vanky, E., Stridsklev, S., Heimstad, R., Romundstad, P., Skogoy, K., Kleggetveit, O., et al. Metformin versus placebo from first trimester to delivery in polycystic ovary syndrome: a randomized, controlled multicenter study*.* J Clin Endocrinol Metab, 2010. 95(12): p. E448-55.

7. Ro, T.B., Ludvigsen, H.V., Carlsen, S.M., and Vanky, E. Growth, body composition and metabolic profile of 8-year-old children exposed to metformin in utero*.* Scand J Clin Lab Invest, 2012. 72(7): p. 570-5.

8. Carlsen, S.M. and Vanky, E. Metformin influence on hormone levels at birth, in PCOS mothers and their newborns*.* Hum Reprod, 2010. 25(3): p. 786-90.

9. Real, F.G., Svanes, C., Omenaas, E.R., Anto, J.M., Plana, E., Janson, C., et al. Menstrual irregularity and asthma and lung function*.* J Allergy Clin Immunol, 2007. 120(3): p. 557-64.

10. Svanes, C., Real, F.G., Gislason, T., Jansson, C., Jogi, R., Norrman, E., et al. Association of asthma and hay fever with irregular menstruation*.* Thorax, 2005. 60(6): p. 445-50.

11. Real, F.G., Svanes, C., Macsali, F., and Omenaas, E.R. Hormonal factors and respiratory health in women--a review*.* Clin Respir J, 2008. 2 Suppl 1: p. 111-9.

12. Polson, D.W., Adams, J., Wadsworth, J., and Franks, S. Polycystic ovaries--a common finding in normal women*.* Lancet, 1988. 1(8590): p. 870-2.

13. Speroff L, Glass RH, and NG, K., *Anovulation and the polycystic ovary*, in *Clinical gynecologic endocrinology and infertility*, M. C, Editor. 1999, Lippincott Williams & Wilkins: Baltimore. p. 487-522.

14. Franks, S. Polycystic ovary syndrome*.* N Engl J Med, 1995. 333(13): p. 853-61.

15. Sheehan, M.T. Polycystic ovarian syndrome: diagnosis and management*.* Clin Med Res, 2004. 2(1): p. 13-27.

16. Davis, W.A.K.M.K.P.G.V.D.T.M.E. Glycemic Exposure Is Associated with Reduced Pulmonary Function in Type 2 Diabetes: The Fremantle Diabetes Study*.* Diabetes Care, 2004. 27(3): p. 752-757.

17. Engstrom, G., Hedblad, B., Nilsson, P., Wollmer, P., Berglund, G., and Janzon, L. Lung function, insulin resistance and incidence of cardiovascular disease: a longitudinal cohort study*.* J Intern Med, 2003. 253(5): p. 574-81.

18. Engstrom, G. and Janzon, L. Risk of developing diabetes is inversely related to lung function: a population-based cohort study*.* Diabet Med, 2002. 19(2): p. 167-70.

19. Lawlor, D.A., Ebrahim, S., and Smith, G.D. Associations of measures of lung function with insulin resistance and Type 2 diabetes: findings from the British Women's Heart and Health Study*.* Diabetologia, 2004. 47(2): p. 195-203.

20. Lazarus, R., Sparrow, D., and Weiss, S.T. Impaired ventilatory function and elevated insulin levels in nondiabetic males: the Normative Aging Study*.* Eur Respir J, 1998. 12(3): p. 635-40.

21. Lazarus, R., Sparrow, D., and Weiss, S.T. Baseline ventilatory function predicts the development of higher levels of fasting insulin and fasting insulin resistance index: the Normative Aging Study*.* Eur Respir J, 1998. 12(3): p. 641-5.

22. Barker, D.J., Godfrey, K.M., Fall, C., Osmond, C., Winter, P.D., and Shaheen, S.O. Relation of birth weight and childhood respiratory infection to adult lung function and death from chronic obstructive airways disease*.* BMJ, 1991. 303(6804): p. 671-5.

23. Haland, G., Carlsen, K.C., Sandvik, L., Devulapalli, C.S., Munthe-Kaas, M.C., Pettersen, M., et al. Reduced lung function at birth and the risk of asthma at 10 years of age*.* N Engl J Med, 2006. 355(16): p. 1682-9.

24. Canoy, D., Pekkanen, J., Elliott, P., Pouta, A., Laitinen, J., Hartikainen, A.L., et al. Early growth and adult respiratory function in men and women followed from the fetal period to adulthood*.* Thorax, 2007. 62(5): p. 396-402.

25. Hancox, R.J., Poulton, R., Greene, J.M., McLachlan, C.R., Pearce, M.S., and Sears, M.R. Associations between birth weight, early childhood weight gain and adult lung function*.* Thorax, 2009. 64(3): p. 228-32.

26. Shi, W., Bellusci, S., and Warburton, D. Lung development and adult lung diseases*.* Chest, 2007. 132(2): p. 651-6.

27. Rotwein, P. Structure, evolution, expression and regulation of insulin-like growth factors I and II*.* Growth Factors, 1991. 5(1): p. 3-18.

28. Ohls, R.K. Erythropoietin and hypoxia inducible factor-1 expression in the mid-trimester human fetus*.* Acta Paediatrica. Supplement, 2002. 91(438): p. 27-30.

29. Wood, W.G. Haemoglobin synthesis during human fetal development*.* British Medical Bulletin, 1976. 32(3): p. 282-7.

30. Tchirikov, M., Kertschanska, S., Sturenberg, H.J., and Schroder, H.J. Liver blood perfusion as a possible instrument for fetal growth regulation*.* Placenta, 2002. 23 Suppl A: p. 153-158.

31. Haugen, G., Hanson, M., Kiserud, T., Crozier, S., Inskip, H., and Godfrey, K. Fetal liver-sparing cardiovascular adaptations linked to mother's slimness and diet. Circ Res, 2005. 96: p. 12-14.

32. Kessler, J., Rasmussen, S., Godfrey, K., Hanson, M., and Kiserud, T. Longitudinal study of umbilical and portal venous blood flow to the fetal liver: low pregnancy weight gain is associated with preferential supply to the fetal left liver lobe*.* Pediatric Research, 2008. 63(3): p. 315-20.

33. Godfrey, K.M., Haugen, G., Kiserud, T., Inskip, H.M., Cooper, C., Harvey, N.C., et al. Fetal liver blood flow distribution: role in human developmental strategy to prioritize fat deposition versus brain development*.* PLoS One, 2012. 7(8): p. e41759.

34. Kiserud, T., Ebbing, C., Kessler, J., and Rasmussen, S. Fetal cardiac output, distribution to the placenta and impact of placental compromise*.* Ultrasound Obstet Gynecol, 2006. 28(2): p. 126-36.

35. Kiserud, T., Rasmussen, S., and Skulstad, S. Blood flow and the degree of shunting through the ductus venosus in the human fetus*.* Am J Obstet Gynecol, 2000. 182(1 Pt 1): p. 147-53.

36. Azpurua, H., Norwitz, E.R., Campbell, K.H., Funai, E.F., Pettker, C.M., Kleine, M., et al. Acceleration/ejection time ratio in the fetal pulmonary artery predicts fetal lung maturity*.* Am J Obstet Gynecol, 2010. 203(1): p. 40 e1-8.

37. Kessler, J., Rasmussen, S., Hanson, M., and Kiserud, T. Longitudinal reference ranges for ductus venosus flow velocities and waveform indices*.* Ultrasound in Obstetrics and Gynecology, 2006. 28(7): p. 890-8.

38. Kessler, J., Rasmussen, S., and Kiserud, T. The left portal vein as an indicator of watershed in the fetal circulation: development during the second half of pregnancy and a suggested method of evaluation*.* Ultrasound in Obstetrics and Gynecology, 2007. 30: p. 757-764.

39. Kessler, J., Rasmussen, S., and Kiserud, T. The fetal portal vein: normal blood flow development during the second half of human pregnancy*.* Ultrasound in Obstetrics and Gynecology, 2007. 30(1): p. 52-60.

40. Ebbing, C., Rasmussen, S., Godfrey, K.M., Hanson, M.A., and Kiserud, T. Hepatic artery hemodynamics suggest operation of a buffer response in the human fetus*.* Reprod Sci, 2008. 15(2): p. 166-78.

41. Kiserud, T., Eik-Nes, S.H., Blaas, H.G., Hellevik, L.R., and Simensen, B. Ductus venosus blood velocity and the umbilical circulation in the seriously growth-retarded fetus*.* Ultrasound Obstet Gynecol, 1994. 4(2): p. 109-114.

42. Kiserud, T., *Venous Hemodynamics*, in *Doppler Ultrasound in Obstetrics and Gynecology*, D. Maulik and SpringerLink (Online service), Editors. 2005, Springer Berlin Heidelberg: Berlin, Heidelberg.

43. Ebbing, C., Rasmussen, S., Godfrey, K.M., Hanson, M.A., and Kiserud, T. Redistribution pattern of fetal liver circulation in intrauterine growth restriction*.* Acta Obstetricia et Gynecologica Scandinavica, 2009. 88(10): p. 1118-23.

44. Kessler, J., Rasmussen, S., Godfrey, K., Hanson, M., and Kiserud, T. Venous liver blood flow and regulation of human fetal growth: evidence from macrosomic fetuses*.* Am J Obstet Gynecol, 2011. 204(5): p. 429 e1-7.

45. Houston, L.E., Odibo, A.O., and Macones, G.A. The safety of obstetrical ultrasound: a review*.* Prenat Diagn, 2009. 29(13): p. 1204-12.

46. Abramowicz, J.S., Kossoff, G., Marsal, K., and Ter Haar, G. Safety Statement, 2000 (reconfirmed 2003). International Society of Ultrasound in Obstetrics and Gynecology (ISUOG)*.* Ultrasound Obstet Gynecol, 2003. 21(1): p. 100.
