# Supplementary material for: Metformin exposure, maternal PCOS status and fetal venous liver circulation: A randomized, placebo-controlled study
Source: PLoS One. 2022 Jan 28;17(1):e0262987. doi: 10.1371/journal.pone.0262987 (PMC8797196; doi:10.1371/journal.pone.0262987)
Supplement: S2 File — (PDF) [file pone.0262987.s003.pdf]

Metformin treatment of pregnant PCOS women and prevention of preterm birth

The PregMet 2 Study

EudraCT number 2011-002203-15
Version 7

A prospective, randomized, double-blind, multi-centre study, where the possible effect of metformin to prevent late miscarriage and preterm delivery is studied in women with polycystic ovary syndrome (PCOS).

Responsible for the protocol:

Tone Shetelig Løvvik MD

Sven M. Carlsen MD, Professor

Eszter Vanky MD PhD

Index:

Index: ... 2

Summary ... 5

Background ... 5

Aim ... 5

Material and methods ... 6

Introduction ... 6

PCOS ... 6

Preterm birth ... 7

Metformin ... 7

The Pilot study ... 7

The PregMet 1 Study ... 7

Table 1. The PregMet 1 Study: Primary endpoint results ... 8

Preterm birth ... 8

Pooled data and per protocol analyses ... 11

Table 2. Per protocol analyses of the Pilot and PregMet 1 studies ... 12

Costs in suffering ... 13

Cost versus benefit with metformin treatment ... 14

Aim of the PregMet 2 study ... 16

Hypothesis ... 16

Definitions ... 16

Oligo-amenorrea ... 16

Hyperandrogenism ... 16

Polycystic ovaries by ultrasound ... 17

Polycystic ovary (PCO) ... 17

PCOS: at least 2 out of 3 criteria ... 17

Gestational age: ... 18

Term date: ... 18

Late miscarriage: ... 18

Preterm birth: ... 18

Hypertension in pregnancy: ... 18

Preeclampsia: ... 19

Gestational diabetes mellitus: ... 19

The endpoints of the study ... 19

Primary endpoint ... 19

Secondary endpoints *19*
Tertiary endpoints *20*

Methods **21**
Design *21*
Participating centres *21*
Inclusion criteria *21*
Exclusion criteria *22*
Study medication *22*
Patients included in the PregMet 2 Study *23*
Pre-inclusion evaluation - screening *24*
1st pre-scheduled visit: *25*
2nd pre-scheduled visit: *26*
3rd pre-scheduled visit: *27*
4th pre-scheduled visit: *27*
5th pre-scheduled visit: *28*
At delivery *28*
Serum analyses *29*

Study duration **30**

"Drop outs" **31**

Withdrawal from the study **31**

Adherence **32**

Variables **32**
Height *32*
Weight *32*
Fasting plasma glucose and OGTT *33*
The Doppler examination of the uterine artery *33*
Ferriman Gallwey score *33*

Randomization **34**

Interim analyses **34**
Blinding **34**

Unblinding **35**

Side effects **35**

Adverse events **36**
Definition *36*
Adverse event (AE) *36*
Serious adverse events (SAE) *36*
SAE not to be reported *36*
SUSAR *37*

Discontinuation of the trial **39**

Concomitant medication **40**

Data collection and management **40**

Management of the study **41**

Study responsibility **43**

Statistical analyses **44**
Risk of type I errors *44*
Modes of testing *45*
Subgroup analysis *45*

Power calculation **45**

Insurance **46**

Our partners – investigators at other study centres **46**

Time schedule **47**

Equipment **48**

Personnel **48**

Economics/Budget **48**

Good Clinical Practice (GCP) **48**

Monitoring **49**

Publication **49**

Access to the material **49**
Ethics **51**

Future plans **52**

Reference List **53**
Appendix I Blood Collection Procedure: **57**
Appendix II, 2-hours glucose tolerance test **59**
Appendix III Ferriman-Galway score **61**
Appendix IV Norwegian labels for study medicine **63**

Summary

Background

PCOS is a common condition among women of fertile age. It can affect almost all aspects of life and reproductive health. The prevalence of pregnancy complications, such as gestational diabetes, preeclampsia, miscarriage and preterm birth seems to be increased among PCOS women. The prevalence of PCOS among women who have experienced preterm delivery is also increased (30 %), compared to a normal population (31). Data from the control women from the same study indicate a 15% prevalence of PCOS in Norwegian women of fertile age. Pooled data from our former RCTs indicates that metformin treatment in pregnancy might prevent late miscarriages and preterm births (1, 2).

Aim

The main aim of the PregMet 2 Study is to investigate whether metformin prevents late miscarriages and preterm deliveries in PCOS women treated with metformin from first trimester of pregnancy to delivery in a large randomized, controlled, multi-centre trial setting.

Material and methods

We will include 18-45 year old women, with PCOS diagnosed according to Rotterdam criteria and with singleton pregnancies, in the first trimester of pregnancy. The participants will be randomized double blindly to 1000 mg metformin twice daily or placebo, from inclusion to delivery. Five prescheduled visits are planned, where we intend to measure weight, blood pressure, perform ultrasound examinations and draw blood samples and perform oral glucose tolerance tests (OGTT).

Introduction

PCOS

The polycystic ovary syndrome (PCOS) affects some 8 -11% of women in the reproductive age group diagnosed according to National Institute of Health criter ia (NIH) (3-5) and probably up to 15-20 % according to The Rotterdam Consensus criteria.

The cornerstones of PCOS are insulin resistance and hyperandrogenicity, although none is mandatory for the diagnosis according to the Rotterdam criteria (6). PCOS has implications both for fertility and pregnancy outcome, while an increasing body of evidence points to a high prevalence of pregnancy complications (7-11). Hyperinsulinemia and hyperandrogenism have been suggested as pathogenic factors in both PCOS and pregnancy complications (12-14) .

Preterm birth

Data on women who had experienced preterm delivery in the county of Namdalen (Namsos hospital) shows a PCOS prevalence of 30 % (ref). The PCOS prevalence among controls who had given birth at term after an uncomplicated pregnancy was 15%.

Metformin

Metformin, an insulin-sensitizer used in the treatment of type 2 diabetes, reduces fasting insulin and testosterone levels in non-obese, non-pregnant PCOS women (15). During the last decade, several retrospective and non-randomized studies have reported beneficial effects of metformin on pregnancy loss and pregnancy complications, in particular gestational diabetes mellitus (GDM) (7, 11, 16-21).

The Pilot study

Our research group performed a pilot study on metfomin treatment of pregnant PCOS women in 2000 - 2003. In this pilot study 7 out of 22 placebo-treated PCOS women experienced serious pregnancy complications, while none of the 18 metformin-treated women had such complications. These were promising results.

The PregMet 1 Study

To confirm these results, a Norwegian multicentre trial, the PregMet 1 Study, was conducted by Eszter Vanky as the principal investigator. However, metformin treatment from the first trimester to delivery, did not influence the incidence of gestational diabetes mellitus (GDM),

preeclampsia (PE), preterm birth (gestational week 22-37), or the composite of these three end points when analysing the results according to *"intention to treat principal"* (2).

Table 1. The PregMet 1 Study: Primary endpoint results

	Metformin N = 135	Placebo N = 135	95% CI	Pvalue
Preeclampsia	10/135 (7.4)	5/135 (3.7)	-1.7 to 9.2	0.18
Preterm delivery	5/135 (3.7)	11/135 (8.2)	-10.1 to 1.2	0.12
New GDM	22/125 (17.6)	21/124 (16.9)	-8.6 to 10.2	0.87
Composite endpoint	35/135 (25.9)	33/135 (24.4)	-8.9 to 11.3	0.78

An editorial in Journal of Clinical Endocrinology and Metabolism accompanying the publication of the PregMet 1 Study stated that:

"There is no study of metformin during pregnancy in women with PCOS that even comes close to this one in terms of scope and quality. This is today the alpha and the omega of metformin use in pregnancy in women with PCOS, the best level I evidence we have to counsel our patients…."(22)

Preterm birth

The WHO defines preterm labour as labour occurring before 37 weeks of pregnancy or before 259 days of pregnancy (A). As prognosis depends on gestational age at birth, we further subdivide preterm deliveries into following groups (23):

- Late preterm birth 34 - 36^{+6} weeks
- Moderately preterm birth 32 - 33^{+6} weeks
- Very preterm birth 28 - 32^{+6} weeks
- Extremely preterm birth < 28 weeks

About 10% of preterm deliveries are extremely preterm, another 10 % very preterm and approximately 80 % moderately or late preterm deliveries.

The obstetric precursors leading to preterm birth are:

1. Delivery for maternal or fetal indications, in which labor is either induced or the infant is delivered by prelabor caesarean section;
2. Spontaneous preterm labor with intact membranes; and
3. Preterm premature rupture of the membranes (PPROM), irrespective of whether delivery is vaginal or by caesarean section (24).

Figure 1 (25, 26).

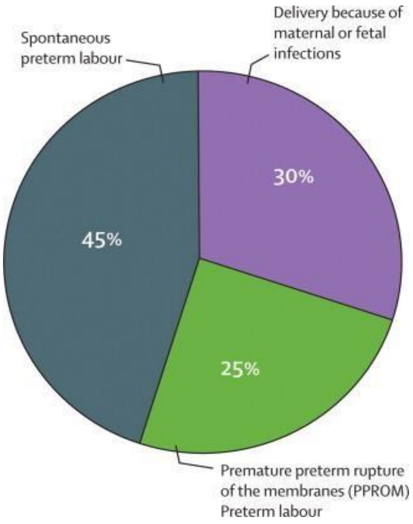

Preterm delivery is a major contributor to neonatal morbidity and mortality (27). Epidemiological risk factors such as socioeconomic status and ethnicity seems to be important (26). Infections, uterine distension and anomaly are other known factors related to

preterm birth. However, the causes of preterm delivery are still essentially unknown and seem to be multifactorial (25).

Former preterm delivery is a significant risk factor. However, almost 50 % of preterm births occur without any identified cause or risk factor (25). The incidence of preterm birth is rising in most western countries, Norway included (28). This occurs despite all attempts to treat and prevent preterm birth.

The fact that former preterm labour is a strong risk factor, indicate a genetic or otherwise individual disposition. And the more or less universal rise in preterm birth rates in the Western world indicates that environmental or lifestyle risk factor changes are involved
Incidence of preterm labour:

Incidence in Norway (B) :	1980: 5.6 %
	1995: 7.5 %
	1999: 7.9 %
Incidence in the USA (C):	1980: 8 %
	1995: 12 %

Although most preterm babies survive, they are at increased risk of neuro-developmental impairments, respiratory and gastrointestinal complications. Preterm infants are also of increased risk of developing cognitive and behavioral abnormalities and of achieving poorly in educational situations. These problems tend to last until adulthood (29). Recent evidence also indicate that infants born close to term (gestational week 34-36) carry a risk of increased morbidity and mortality risk, compared with term infants(E).

There are major socioeconomic costs connected to these patients. There are obvious costs due to initial neonatal treatment. But even more substantial is the health service costs following

discharge from the neonatal unit. This also represents long-term burdens, on affected families, but also on health, education and social services (30).

Recently published data on women who had at least one preterm birth, showed a PCOS prevalence of 25%, while 14% had PCOS among a control group of women who had given birth at term after an uncomplicated pregnancy (31). These data support former reports on preterm deliveries occurring more often among PCOS women and points to the fact that there is an increased prevalence of PCOS among women who experienced preterm birth.

Pooled data and per protocol analyses

In the PregMet 1 Study we found that, according to *"intention to treat"* analysis,
5 out of 135 metformin-treated and 11 out of 135 placebo-treated PCOS women experienced preterm birth (p=0.12).

However,

- Biologically and medically, late miscarriage (gestational week 13-21) and preterm birth is a "continuum", not separate conditions. Adding the miscarriage and preterm data in the PregMet 1 Study and analyzing it according to *"intention to treat"* principle, we find 13 late miscarriages/preterm births in the placebo group (N=135) and 5 in the metformin group (N=135), (p=0.055).

- If we pool the results from the Pilot Study and the PregMet 1 study, we have 18 of 157 (11.5%) late miscarriages/preterm births in the placebo group and 5 of 153 (3.3%) in the metformin group (p=0.01).

- In *”per protocol”* analysis including only women with good drug compliance (drugs taken as intended or 1-2 tablets for a maximum of 4 weeks or no tablets for a maximum of 2 weeks; 84%) of the PregMet 1 Study, 3 out of 108 (2.8%) metformintreated and 11 out of 108 (10.2%) placebo-treated women delivered preterm, p = 0.04.
- Adding the miscarriage and preterm data from the Pilot study to the PregMet 1 Study, and analyzing it *“per protocol”* we find that 18 of 129 (14.0%) women in the placebo group and 3 out of 125 (2.4%) in the metformin group had either late miscarriage or preterm birth, i.e. < 37 gestational week (p = 0.001). This yields a number needed to treat (NNT) = 8.7 to prevent one late miscarriage or preterm birth.

Table 2. Per protocol analyses of the Pilot and PregMet 1 studies

Placebo		Metformin N = 129	N = 125
Gestational age in weeks at miscarriage or delivery			
Pilot study	PregMet 1 Study	Pilot study	PregMet 1 Study
21	17	None	35
29	21		36
31	30		36
36	31		
36	32		
	32		
	34		
	34		
	34		
	34		
	36		
	36		
	36		

Costs in suffering

Both short and long-term costs in money and sufferings of preterm birth are immense. Although preterm babies born at week 35-37 can be a medical challenge, the real suffering for the patients and relatives, and the costs for the society are often the babies born < gestational week 35. In the present material, 12 out of 18 preterm deliveries and late miscarriages in the

placebo group, were born < gestational week 35. None were born < 35 gestational weeks among metformin treated women taking their medication as prescribed.

The treatment costs (from delivery to discharge) for a preterm baby in average are (F)

< 1000 g (< 27 weeks)	770 000 NOK
1000g – 1500g (around 27-30 weeks)	575 000 NOK
1500g – 2500g (around 30 – 35 weeks)	240 000 NOK (36)

None of the preterm babies in the metformin-group had birth weight < 2500g, while 9 babies in the placebo group had a birth weight between 1500g – 2500g. The initial treatment cost for these neonates was around 2.2 million NOK. Later costs for treatment of associated conditions and diseases, pedagogic support etc. adds to these initial costs. For example, tuition costs/pedagogic support for a multi-handicapped pupil at grade 11, is 700000 NOK/year and for a handicapped pupil with individual learning goals is 400000 NOK/year (South Trøndelag county 2010)

Cost versus benefit with metformin treatment

To treat one pregnant PCOS woman with metformin 1000 mg x 2 daily from gestational week 8 to 37 costs 100 Euro. To prevent one preterm birth or late miscarriage would cost 870 Euro, about 7000 NOK. If we have conservative estimates of the effect used in the power calculation, we find that 20 women have to be treated to prevent one preterm birth. That would be 2 000 Euro or 16 000 NOK. This equals 6-7% of the immediate costs of one preterm baby.

Accordingly, the above described treatment would, if it can be verified in the PregMet 2 Study, probably be among the most cost effective, preventive treatments ever. Actually, metformin is so cheap, old and safe that the pharmaceutical industry does not support research on its effects. This matter has been addressed and discussed at international meetings and congresses and is regarded as a problem.

These results from the PregMet 1 Study and the sub analyses presented above on preterm birth were presented at the NFOG (Nordic Federation of Societies of Obstetrics and Gynecology) PCOS meeting in Copenhagen in November 2010. There was a great interest and enthusiasm for the possible effect of metformin on preterm birth and a call for an international multicentre study to settle this important clinical question as soon as possible.

Aim of the PregMet 2 study

The overall aim and primary end point of the PregMet 2 Study is to investigate whether metformin prevents late miscarriages and preterm deliveries in PCOS women treated with metformin from first trimester of pregnancy to delivery in a large, randomized, controlled, multi-centre trial setting.

Hypothesis

Metformin compared to placebo treatment from the first trimester to term, reduces the prevalence of late miscarriage (gestational week 13-22) and preterm birth (gestational week < 37) in PCOS women diagnosed according to Rotterdam 2003 consensus criteria, with singleton pregnancy.

Definitions

Oligo-amenorrea

Defined as a cycle length of ≥ 35 days or **<** 10 per year.

Hyperandrogenism

- Biochemical hyperandrogenemia:
  (s-testosterone ≥ reference value for women at each study site for the time being and/or s- SHBG ≤ 30 nmol/L).

- Clinical hirsutism: Ferriman-Gallwey score > 6

Polycystic ovaries by ultrasound

Defined as at least 12 follicles in one ovary and/or an ovarian volume ≥ 10 ml (volume = $0.52 \cdot d^1 \cdot d^2 \cdot d^3$) in at least one ovary.
If there is a follicle ≥ 10 mm, repeat ultrasound at the time of ovarian quiescence

Polycystic ovary (PCO)

□ 12 or more follicles in the ovary, each follicle measuring 2-9 mm in diameter <u>and/or</u>

PCOS: at least 2 out of 3 criteria

1. PCO in at least one ovary
2. Oligo-/anovulation (clinically diagnosed as oligo-/amenorrhea, i.e. menstrual cycles > 35 days or <10 menstruations/year
3. Hyperandrogenism; clinical or biochemical: Biochemical hyperandrogenemia (stestosterone above upper reference value at the actual laboratory used) and/or clinical hirsutism (defined in this study as weekly Ferriman-Gallway score ≥ 6)

Exclusion of: Congenital adrenal hyperplasia (CAH), non-classical CAH, Cushing disease, acromegaly and androgen secreting tumors.

Gestational age:

Gestational age is determined in the first trimester by a vaginal ultrasound examination. Crown-rump-length (CRL) and/or bi-parietal diameter (BPD) are measured.

Term date:

Term date is determined by a mid-second trimester, trans-abdominal ultrasound examination measuring BPD, mean abdominal diameter (MAD) and length of femur, or using the appropriate method that is standard at the actual study site.

Late miscarriage:

Miscarriage occurring between gestational weeks 13 + 0 and 22 + 6.

Preterm birth:

Spontaneous or operative birth occurring before gestational week 37 + 0.

Hypertension in pregnancy:

Blood pressure measured after at least 10 minutes of comfortable chair rest ≥140/90 mmHg at two different occasions without proteinuria or other signs of preeclampsia occurring after week 20 in pregnancy.

Preeclampsia:

Blood pressure measured after at least 10 minutes of comfortable chair rest ≥140/90 mm Hg at two different occasions and proteinuria (1+ protein in two different occasions or 2+ protein in one occasion on a urinary dip stick).

Gestational diabetes mellitus:

Gestational diabetes mellitus (GDM) is defined as a fasting plasma glucose ≥7.0 mmol/L or a 2 hour plasma glucose ≥ 7.8 mmol/L during a standard 75 g per oral glucose tolerance test (OGTT) performed according to the WHO standard.

Hirsutism:

Ferriman – Gallwey score ≥6

The endpoints of the study

Primary endpoint

- The combined incidence of late miscarriages and preterm births. This includes spontaneous births, induced vaginal deliveries and operative deliveries on medical indications.

Secondary endpoints

- No. of patients treated with vaginal progesterone indicated by short cervix as a sign of imminent preterm delivery

- NICU admissions and total number of days in NICU/baby
- No. of patients hospitalized (except delivery and post partum)
- Total number of hospitalization days/ hospitalized participant
- The incidence of gestational diabetes mellitus
- The prevalence of :
  - ○ Preeclampsia ○

    Hypertension in pregnancy
- Vaginal bleeding in pregnancy

Tertiary endpoints

- Safety of treatment with metformin
- Weight gain in pregnancy
- Newborn data
- Birth weight
- Birth length
- Head circumference
- Apgar score/
- pH and base excess(BE) from umbilical cord blood

- Malformations

Methods

Design

Prospective, randomized, double-blind, Nordic multi-centre study

Participating centres

Public or private, secondary or tertiary clinics dealing with gynaecology and obstetrics. We will invite centres form the Nordic countries. All principal investigators in all study centres have to be specialists in obstetrics/gynecology or under on-going specialist-training.

Inclusion criteria

- Age: 18-45 years
- PCOS diagnosed according to Rotterdam 2003 criteria,
- Mode of conception: Any
- Single, viable fetus, diagnosed by vaginal ultrasound
- Gestational week at inclusion: week 6 + 0 to 12 + 6.
- Wash out for metformin: at least 7 days
- Able to communicate fluently in the official language at the study cite or English

- Informed consent

Exclusion criteria

- Gestational age > 12 weeks+6days
- Any type of diabetes (except GDM in former pregnancy).
- Known liver disease or ALAT > 100 IU/L
- Known renal disease, renal dysfunction or serum creatinine > 110 µmol/L .
- Acute conditions that may worsen renal function, such as dehydration, septicaemia, cardiovascular collapse (shock), and intravascular administration of iodinated contrast.
- Acute or chronic conditions that can lead to tissue hypoxia, such as cardiac or respiratory failure, recent myocardial infarction or shock.
- Known hypersensitivity to any component of metformin
- Breastfeeding
- Known alcohol or drug abuse
- Use of drugs interfering with metformin: erytromycin (or other macrolides), cimetidin, anticoagulation therapy
- Unsuitable for participation of any other reasons

Study medication

- Metformin 500 mg x 2 daily the first week and then 1000 mg x 2 daily from the second week to delivery.

- Placebo 1 tablet x 2 daily the first week. And then 2 tablets x 2 daily from the second week to delivery.
- To counteract a possible metformin action on folic acid and vitamin B_{12} levels, patients will be advised to take the daily dose of folic acid recommended in the country of the study centre. Further, one daily multivitamin tablet containing vitamin B_6 and B_{12} will be recommended.
- Both metformin and placebo are produced by Weifa A/S. The tablets will be packed into boxes at the Hospital Pharmacy in Trondheim. After randomization the medication will be sent to cooperating pharmacies at the study site.
- The Hospital Pharmacy in Trondheim is responsible for medical accounting in the trial.
- A separate drugaccountibility log will be registered at each site.

Patients included in the PregMet 2 Study

- The overall intention and philosophy of the trial, is that included patients should receive the recommended and appropriate treatment available at each country and centre.
- Participation in the study does not limit any kind of diagnostic evaluation, examination or treatment option regarded as necessary during pregnancy.
- The participants in the PregMet 2 study can however not participate in other medical trials during the actual pregnancy.

- Patients can only be included in the PregMet 2 Study once.

Progesterone treatment to prevent preterm delivery

In Iceland vaginal progesterone administration is used to prevent preterm delivery according to national guidelines;

1. In multiparous women who had former preterm delivery and
2. In women where cervix is found to be < 15 mm before gestational week 32. There are however no screening program for cervix length for asymptomatic women.

These treatment regimens interfere with the primary endpoint of the PregMet study. Therefore to avoid protocol violation and ethical conflict we decide the following:

1. At study centres in countries where progesterone is used prophylactic in multiparous women with former preterm birth, only nulliparous women will be included in the study.
2. In women with signs or symptoms of imminent preterm delivery, the routine treatment at the study centre should be given, - included vaginal progesterone treatment.
3. Vaginal progesterone treatment in such cases of imminent preterm delivery will be registered as a secondary endpoint.

If the progesterone treatment routine is introduced in other participating countries during the on-going study, these countries will adhere to the instructions above.

Pre-inclusion evaluation - screening

- Information (verbal and written to the potential participating woman).
- Signed written informed consent.

 Confirming the diagnosis of PCOS; anamnestically and from patient records

- Vaginal ultrasound examination to verify intrauterine viable singleton fetus (visualised heart beats), gestational age < 12+6 weeks. (CRL < 65 mm or BPD < 23 mm
- Blood tests:
- fasting plasma or serum glucose,
- serum ALAT
- serum creatinine

Standardized interviewer-administered CRF will be used to obtain self-reported data on former medical and gynecologic/obstetric history, ethnicity, and employment, smoking habits, study medication and concomitant medication. Biometric variables, including height (measured only at inclusion), weight, blood pressure, and heart rate, are recorded at inclusion and at each pre-scheduled visits at gestational week 19, 28, 32 and 36.

1st pre-scheduled visit:

Inclusion gestational age ≤ 12^{+6} weeks

- Written general diet and lifestyle information recommended to pregnant women from the national health authorities in each country will be given.
- Anamnestic data
- Physical examination
- Blood pressure

- Weight and height

  Breast-size estimation
- Hirsutism score (http://www.hirsutism.com/hirsutism-biology/ferriman-gallweyscore.shtml)
- Vaginal ultrasound examination:
- Visualized heart activity
- CRL and /or BPD measured
- Description of the ovaries
- Fasting blood samples (both serum and full blood) for freezing
- 2 hour OGTT (Fasting and 2h serum glucose to be measured locally)
- Questionnaire on depression EPDS (standard questionnaire used in and after pregnancy in routine pregnancy follow-up)

2nd pre-scheduled visit:

Gestational age 19 ± 1 week

- Anamnestic data
- Physical examination
- Blood pressure
- Weight
- Ultrasound examination:
- Mid-pregnancy determination of term

- Doppler measurement of the uterine artery (OPTIONAL)

- Fasting blood sample (both serum and full blood) for freezing
- Fasting blood sample for glucose

3rd pre-scheduled visit:

Gestational age 28 ± 1 weeks

- Anamnestic data
- Physical examination
- Blood pressure
- Weight
- Ultrasound examination (Doppler) OPTIONAL
- 2 hour OGTT (Fasting and 2h serum glucose to be measured locally)
- Blood samples (both serum and full blood) for freezing
- Questionnaire on depression EPDS (standard questionnaire used in and after pregnancy in routine pregnancy follow-up)

4th pre-scheduled visit:

Gestational age 32 ± 1 weeks

- Anamnestic data
- Physical examination ○ Blood pressure

  - Weight

- Ultrasound examination:
  - Fetometry
- Fasting blood samples (both serum and full blood) for freezing
- Fasting blood sample for glucose

5th pre-scheduled visit:

Gestational age 36 ± 1 weeks

- Anamnestic data
- Physical examination ○ Blood pressure ○ Weight
  - Breast-size estimation
- Fasting blood samples (both serum and full blood) for freezer
- Fasting blood sample for glucose

At delivery

- Collecting maternal data from the delivery and clinical data from the newborn
- Sampling of arterial and venous cord blood immediately after birth (OPTIONAL)
- Sampling of placenta biopsies (OPTIONAL)

At 8-weeks postpartum: Structured telephone interview on

- Birth experiences
- Breastfeeding experiences
- Questionnaires on postpartum depression
- Complications postpartum: infections, deep venous thrombosis, lung embolism, mastitis, other serious adverse events
- New born hospitalization, illness, anomalies
- Left over study medication reported
- Questionnaire on depression EPDS (standard questionnaire used in and after pregnancy in routine pregnancy follow-up)

Blood samples

All blood samples will be collected in the morning between 08 and 11, and after an overnight fast. All screening analyses will be analysed at the local hospital.
Ten (10) test tubes of whole blood from each patient are centrifuged after 20 minutes, and serum is filled in nine (9) cryotubes with at least 1.8 ml of serum in each, and frozen at – 70 º C (or – 80 º C if that is preferred).Four EDTA tubes with whole blood will be filled, turned up and down a couple of times and transferred into cryotubes and freezed.

Two tubes for “buffy coat” will be separately centrifuged, processed and freezed.

All cryotubes will be marked with a unique barcode. The tubes will be marked with a colour code for each visit. (Red hat for inclusion, yellow in week 19, blue in week 28, green in week 32, brown in week 36.

Umbilical cord blood will be sampled at delivery (optional). We aim to have both venous whole blood samples, and both arterial and venous serum samples (Handled as described above). These will be marked with a purple hat.

All serum and whole blood will be sent to: Tone Shetelig Løvvik/ or study midwife, Fødeavdelingen, Kvinneklinikken, St. Olavs Hospital, 7006 Trondheim, Norway (phone +47 72575715 E-mail tone.shetelig.lovvik@stolav.no). Ahead of shipment the receiver should be contacted. No shipment is to be done on Thursdays, Fridays or the last two days before a public holidays.

Study duration

For each woman the participation in the present study will last from inclusion till 8 weeks post partum. The study medication will be stopped at delivery, but data will be collected and adverse events reported until the 8th week post partum.

"Drop outs"

Data will primarily be analyzed according to the "intention to treat" principle, therefore data from patients who stop medication or do not present at pre-scheduled visits will also be included in the data analysis.

Clarification:

«Drop-out»: is a general term for those patients included in a study who

- Clearly state that they do not want to continue in the study, (-they will not meet at pre-scheduled visits in the study) OR
- Clearly state that they do not intend to take any more study medication OR
- Clearly state that they do not want any contact with the study investigators

NOT "Drop-out": patients who

- Miss/forget an appointment, but attend other visits
- Reduce or do not take study medication because of side effects Forget to take their study medication, but intend to take it.

In our "intention-to-treat analyses" we will include data from all "drop-outs". This is the meaning of "intention-to-treat".

Data included from drop-outs are:

- already collected data and samples and
- Information from delivery mother/baby. (The informed consent has not been withdrawn)

- **Already collected data will not be destroyed or deleted routinely**, but if the patient explicitly asks for it (withdraws informed consent); we will delete it and not collect information from her delivery and baby at delivery.

Withdrawal from the study

Patients may withdraw from the trial at any time. If a patient withdraws from the trial she will be asked if the material already collected and information about the future delivery can be collected and included in the trial data base. If the patient requests, all information about the patient will be withdrawn. This is described in the informed consent. We wish to include and follow 1000 women in our trial. In our former studies withdrawal was a very minor problem, thus we expect a similar pattern in this trial, indicating no substitute is necessary. If this

proves to be a problem in accordance to keep our desired sample size, we might open up to include additional patients as long as the inclusion period is not stopped.

Adherence

Adherence will be evaluated by asking the participants about their tablet intake in a systematic manner at each visit. In addition participants will be asked to count and report their remaining tablets in connection with the telephone interview at 8 weeks postpartum. Further monitoring of the number of tablets is not required.

Good adherence is defined as consumption of ≥ 90% of the estimated drug dose during the study period. Acceptable compliance will be defined as drug consumption ≥ 70% and < 90%. Poor compliance is defined as < 70% drug consumption.

Variables

Height

Will be measured in cm. Measured without shoes, once – at the inclusion visit

Weight

Weight will be measured at each prescheduled visit, without shoes and with light clothes on. Weight in early pregnancy and weight development through pregnancy is an important factor in almost all conditions in pregnancy.. Weight will be measured and approximated to the nearest kg.

Fasting plasma glucose and OGTT

The prevalence of gestational diabetes is high among PCOS. It is important to identify impaired glucose tolerance and GDM as early as possible in pregnancy and treat it appropriately

The Doppler examination of the uterine artery

Up to date ultrasound devices in daily practice will be used. Doppler ultrasound will be measured by abdominal approach. The point where the uterine artery crosses the internal iliac artery will be identified and the gate will be placed within 1 cm on each side of this point. The angle will be kept below 30 degrees. Pulsatility index (PI) will be measured on the right and left side 3 times. The lowest PI (LPI) for each side will be used in further analyses. Presence of a diastolic notch will be registered on each side.

Ferriman Gallwey score

The Ferriman-Gallwey score is scored according to established standards

(http://www.hirsutism.com/hirsutism-biology/ferriman-gallwey-score.shtml)

To identify hirsutism in a more structured manner. See appendix.

Breast volume estimation

The volume of the breasts will be estimated by specially designed cups. This will be done at inclusion and at the last visit in pregnancy. See appendix.

Randomization

Randomization will be performed at the Unit for Applied Clinical Research (AKF), Department of Cancer Research and Molecular Medicine, Norwegian University of Science and Technology, Trondheim, Norway. Randomization will be in blocks of ten (5 metformin and 5 placebos) for each country. An unknown sized block will be introduced at the beginning of the inclusion in each country to make it impossible to estimate the actual study allocation of the participants.

Interim analyses

No interim analyses will be performed.

Blinding

The investigators at the study centers will enter patients into the web CRF at Unit for Applied Clinical Research. The participants then get a unique CRF number. The national pharmacy distributing the study medication will send the study medication to the including center. The study medication will be handed out by the investigator. When the participant is randomized, she will get a randomization number. The study drug package will have the same number as the patients randomization number.

The participants and care providers will be blinded for treatment allocation.

Unblinding

In a case of emergency unblinding or breaking the codes might be necessary. The sponsor/Dr. med. Eszter Vanky shall be informed as soon as possible. The code log will at all times be kept at the Hospital Pharmacy in Trondheim. Necessary unblinding can be done by the pharmacist on call at the St.Olavs Hospital.

Phone number: + 47 815 55 850

This information is found in the site file at each study center.

.

Side effects

The most common side effects are gastrointestinal discomfort. Gastrointestinal side effects, headache and dizziness will be registered in a structured form in the CRF. Other possible and suspected side effects will be documented.

In case of suspected unacceptable side effects, the study medicine dosage will be adjusted or stopped.

All medication used by the participants will be registered.

Adverse events

Definition

Adverse event (AE)

An adverse event (AE) is defined as any untoward medical occurrence in a patient or clinical investigation subject administered a pharmaceutical product, which does not necessarily have a causal relationship with this treatment. An AE can therefore be any unfavourable and

unintended sign (including an abnormal laboratory finding), symptom, or disease temporarily associated with the use of a medical product, whether or not related to the medical product.

Serious adverse events (SAE)

Any serious adverse events (SAE) arising during the study must be reported to the principal investigator of the study within 24 hours. SAE is defined as any untoward medical occurrence that at any dose:

- Results in death,
- Is life-threatening (i.e. the patient was at risk of death at the time of the event. It does not refer to an event, which hypothetically might have caused death if it was more severe).
- Requires in-patient hospitalisation or prolongation of existing hospitalisation.

SAE not to be reported;

Conditions to be reported as study endpoints, **and not as SAE**, even if they are leading to hospitalization.

- Gestational diabetes
- Preeclampsia and severe hypertonia
- Preterm delivery
- Miscarriage
- Preterm rupture of membranes
- Severe intrauterine growth restriction

- Severe bleeding in pregnancy

In the case of an SAE please refer to: Principal investigator, Dr.med Eszter Vanky, St.Olavs Hospital, Trondheim,

Phone office: +47 72 57 38 19

Mobile phone + 47 996 18 028

Study mobile +47 977 98 745

E-mail: eszter.vanky@ntnu.no

SUSAR (Suspected Unexpected Serious Adverse Reactions)

According to “Regulation relating to clinical trials on medicinal products for human use”, the sponsor shall ensure that all suspected adverse reactions occurring in connection with a clinical trial of drugs for human beings that are fatal or life-threatening, and which are unexpected, are reported. The same is applicable for suspected adverse reactions that are serious and unexpected

All SUSARs in Norway shall be reported individually to the Norwegian Medical Agency and to EMEA by the sponsor, Dr.med Eszter Vanky, within 7 days for fatal or life-threatening SUSARS and within 15 days for other serious and unexpected adverse events. The principal investigator at each site in Norway shall report all SUSARs to the sponsor within 24 hours. In the other Nordic countries the SUSARs shall be reported to the national principal investigator within 24 hours. The national principal investigator will report to the medicine agency in their country according to their regulations and to the sponsor,Dr.med Eszter Vanky. SUSARs

recorded outside Norway will be reported to the Norwegian Medical Agency once a year. The following conditions *will not be* regarded as adverse effects, and thus they will not be reported as AE/ SAE or SUSARs:

a) frequently occurring minor conditions in pregnancy

- Fatigue
- Pelvic girdle pain/low back pain
- Uncomplicated, transient dizziness
- Uncomplicated transient headache
- Edema
- Vaginal yeast infection (candidiasis)
- Varicose veins in the legs and vulva/vagina
- Haemorrhoids
- Uncomplicated urinary tract infection (Group-B-streptococcus in the urine, must be registered as AE)

b) Adverse events **not to be registered** until 8 weeks post partum frequently occurring minor conditions in **post partum** until 8 weeks

- Uncomplicated, transient dizziness
- Uncomplicated transient headache
- Vaginal yeast infection (candidiasis)
- Varicose veins in the legs and vulva/vagina
- Haemorrhoids

- Minor, transient problems related to the vagina/perineum (itching, soreness, protracted minor bleeding after delivery, minor urinary incontinence)
- Local infection, soreness, opening of episiotomy, laceration or operation scar after CS
- Problems with breastfeeding, sore breast (these are reported elsewhere in the CRF.

c) Offspring

All hospitalization must be reported and sustained colic (not crying a few nights)

Not reported as AE:

- Minor cold
- Problems to suckle /take breast
- Minor infections in the eyes/ umbilicus
- Stiff neck

Discontinuation of the trial

In case of unexpected emergencies, security problems etc the sponsor might want to discontinue the trial as a whole. If the sponsor discontinues the clinical development of an investigational product (i.e. for any or all indications, routes of administration, or dosage forms), the sponsor should maintain all sponsor-specific essential documents for at least 2 years after formal discontinuation or in conformance with the applicable regulatory requirement(s). If the sponsor discontinues the clinical development of an investigational product, the sponsor should notify all the trial investigators/institutions and all the regulatory authorities.

Concomitant medication

Concomitant medication will be registered by generic name and treatment duration.

Medications taken for frequently occurring minor conditions in pregnancy (listed above) will not be registered.

The participants should not take any other vitamins, trace elements, dietary supplements or herbal medicine as long as they participate in the study. Fish oil and omega 3 can be taken

Data collection and management

Data collection and management will be performed by the internet based solution (web-CRF) at the Unit for Applied Clinical Research (AKF) in Trondheim. All women who sign the consent of participation and are screened for participation will be allocated a CRF number in an electronic clinical research form (CRF). Those who are randomized will also have a randomization number. The principal investigator in charge at each study centre keeps a code log with the CRF number allocated to each study participant. A copy of this code log will be kept by the Principal Investigator at each study site.

The web-CRF will be structured and will be completed at each consultation. Original patient files and results from blood tests will be filed for at least 15 years at each study site.

In the database (web-CRF) the patient will only be registered by a CRF number, initials (the first two letters of the given name and the first letter of the family name; ex: Anne Pettersen=ANP), age at inclusion and study site.

Management of the study

The study will be performed according to a "penta-blind" approach.

1. The patient will be blinded for the study medication.
2. The investigator will be blinded for treatment allocation. This is the traditional "double-blind" design and represents the gold standard of pharmaceutical research; randomised double-blinded studies.
3. Obstetricians who evaluate the end points (quality check the given diagnoses) of the study will not be among those who have consulted or treated of the participants during the study. To minimize the chance to estimate the treatment allocation of the individual patient, those who evaluate end points, will only be given access to the data they need to classify the outcomes, adverse events and safety data. When this is done and the monitoring is finished the data base will be locked.
4. All statistical procedures will be performed by a statistician blinded for the actual medication; groups will be analysed only as group 1 and group 2.
5. All results presented in tables and figures, which will be part of the first major publication from the PregMet 2 Study, will be worked out before the codes are broken.

   This blinding procedure will be documented at the Unit for Applied Clinical Research (AKF) in Trondheim.

6. Regional forskningsbiobank Midt-Norge is responsible for the PregMet2 study biobank. Responsible for the Regional forskningsbiobank Midt-Norge is professor Jostein Halgunset (leader of the Regional forskningsbiobank Midt-Norge).

Sub-studies of the PregMet2 study

There are 3 sub-studies connected to the PregMet2 study

1. The RespPreg study "Metformin in lung health in pregnant women with PCOS"

   The sub-study has a questionnaire part (run in Bergen, Trondheim and Ålesund) and a spirometri test part (run in Bergen and Trondheim).

   See separate protocol, CRF and patient information/consent.

2. The Polymeter study "Polycystic ovary syndrome metformin elimination research"

   The sub-study collects extra blood samples in pregnancy and post partum. (The substudy is run in Trondheim)

   See separate protocol and patient information/consent.

3. The CircMet study "The impact of Metformin on the fetal pulmonary and hepatic circulation" (run in Bergen)

   This study performs additional ultrasound examinations on the patients

   See separate protocol, CRF and patient information/consent.

Study responsibility

Eszter Vanky

- has the medical responsibility for the study. She is the representative of the sponsor, The Medical Faculty at The Norwegian University of Science and Technology. o will be the principal investigator for the gestational and post partum part of the study.
- she will supervise, help and support Tone Shetelig Løvvik in all aspects and recruit investigators to the study.

Tone Shetelig Løvvik:

- is responsible for the recruitment to the study in Trondheim, include and follow up the participants.
- she will co-ordinate and support other centres.

Sven M. Carlsen

- is the head of the Unit for Applied Clinical Research (NTNU).
- The trial use the internet based randomization and webCRF and will be monitored by the Unit for Applied Clinical research (NTNU) Trondheim as part of building the CTU (Clinical Trial Unit) capacity at AKF. This also includes participation in negotiating and decision making with other study centres and partly supplying manpower and expertise to run the study according to the obligations accepted by the sponsor, The Medical Faculty at The Norwegian University of Science and Technology.
- Unit for Applied Clinical Research will support and administer some of the practical economic issues.

Statistical analyses

Statistical analysis of the data will be performed when all data are collected and quality checked. Data will be analyzed by an experienced statistician at Unit for Applied Clinical Research according to the "pentablind" approach.

The main outcome (late abortions and preterm births) will be the end points by intention to treat (ITT) analyses. However, secondary "per protocol" analysis will also be performed. The metformin group will be compared to the placebo group using non-paired parametric and non-paired non-parametric methods as appropriate. Only two-sided tests will be used.

Risk of type I errors

The probability of a type I error is the probability of rejecting the null hypothesis when the null hypothesis is true. This is commonly referred to as the significance level of a test. Since we consider late miscarriages and preterm delivery to be a continuum, our study has only one primary endpoint. With only one primary endpoint, and a significance level of $p = 0.05$ and two-sided test we consider the risk of type I error extremely small and acceptable.

Modes of testing

After having conducted a virtually identical study on 273 pregnant women with PCOS, there is no reason to expect significantly more late miscarriages or preterm deliveries in the metformin group then in the placebo group. In spite of this we will only perform two-sided testing of our results.

Subgroup analysis

Subgroup analysis will be performed based on PCOS phenotype and pre-pregnant BMI below and above 30 kg/m^2.

Power calculation

Based on the results from pilot and PregMet 1 Study the combined incidence of late miscarriages and preterm birth in women in the control (placebo) arm of the PregMet 2 Study will be at least 10%. Data from the PregMet 1 Study indicate a 60% reduction and data from the pilot study even more. We aim to show a 50% reduction with a power of 85% and alfa = 0.05. To do so, we need to include 500 patients in each treatment group.

Events in controls	Events in metformin	Power to detect an □=0.05	Number needed in each treatment arm
10%	4%	80%	280
10%	5%	80%	430
10%	6%	80%	720

Insurance

The Norwegian participants will be insured by the “Legemiddelansvarsforeningen”.

Participants in other countries will be insured by a similar instance in each country.

Our partners – investigators at other study centres

Dr Tone S. Løvvik	St. Olavs Hospital, Trondheim	Norway
Dr Francisco Real	Haukeland sykehus, Bergen	Norway
Dr Sissel Hjelle	Ålesund Sykehus	Norway
Dr Birgitte Mork	Sykehuset Telemark, Skien	Norway
Dr. Solvieg Thorarinsdottir	Sykehuset Telemark, Skien	Norway

Dr Renata Zabielska	Sykehuset i Vestfold	Norway
Dr. Kristin Voss Hestvold	Drammen sykehus, Vestre Viken	Norway
Prof Inger Sundström Poromaa	Uppsala University Hospital	Sweden
Prof Angelica Linden Hirschberg	Karolinska Sjukhuset, Stockholm	Sweden
Prof Marie Bixo (from 2014-04-01)	University Hospital in Umeå,	Sweden
Dr Izabella Jawad	Universitetssjukhuset i Örebro	Sverige
Dr Johanna Molin (from 2014-04-01)	Södersjukhuset, Stockholm	Sverige
Dr. Berglind Steffensen	University Hospital in Reykjavik	Iceland
Dr Snorri Einarsson	University Hospital in Reykjavik	Iceland

Time schedule

If Tone Shetelig-Løvvik receives a 50% research grants:

May 2011 – March 2012

Working on applications to ○ The

ethical committee ○ Norwegian

Medicines Agency ○ EUDRACT

form

○ Norwegian Research Council

Working on ○ Patient information ○

Recruiting investigators ○ Logistics

for drug administration

Supporting principal investigators in the participating countries ○

Applications to the authorities

April 2012 – January 2013 ○

Study start-up meetings

October 2012 –July 2015 ○ Inclusion and

follow up of the patients ○ Co-ordination

of the trial

2015-2016

Analysing data and working on the main paper from the PregMet 2 study

Equipment

We will use the room facilities and equipment; (weight, ultrasound machines, and gynaecological examination equipment) provided at the hospital at each study centre.

Personnel

We aim to connect a midwife or nurse at each study centre to the PregMet 2 Study.

Economics/Budget

To cover the costs of the present study, we have applied for grants from

○ Forskningsrådet - recieved ○ Samarbeidsorganet – declined. We will apply again ○ Novo Nordisk Forskningsfond- received ○ Nordic Federation of Gynecology and Obstetrics NFOG - recieved

Good Clinical Practice (GCP)

The study will be conducted in full compliance the GCP standards given in the guidelines. The investigators are responsible for conducting the study in full accordance with the clinical study protocol, the latest version of the Declaration of Helsinki, and Good Clinical Practice: http://ec.europa.eu/health/files/eudralex/vol-10/3cc1aen_en.pdf

Monitoring

In Norway personnel from Unit for Applied Clinical research will monitor the study. In the other participating countries separate CRO organizations will be used for monitoring.

- Monitoring will include:
    - All the informed consents
    - All SAE and SUSARs
    - The first three patients will be fully monitored at each study site.
    - The next four patients; inclusion and exclusion criteria will be monitored and the inclusion visit, the visit in week 28 and the report from delivery.
    - A random sample (drawn by the Unit for Applied Clinical Research) of patient visits.

All in all approximately 20 % of the total data in each country will be monitored.

Publication

We aim to publish the results in international medical journals of the highest possible rank. In the main manuscript Tone Shetelig Løvvik is first author, Sven M. Carlsen is second author and Eszter Vanky is last author. Others are co-authors when they fulfil the Vancouver Declaration for authorship. The principal investigators in each country come after the second author, thereafter co-authors are included in an order that reflects the number of patients included at their study site.

Access to the material

Tone Shetelig Løvvik, Sven M. Carlsen and Eszter Vanky will have equal access and possibility to use the data and biological material collected in the study for further research beyond the main paper. When doing so all three will be given possibility to participate in the subprojects to a degree that justifies authorship on the particular sub-project.
Sven M. Carlen will be principal investigator and responsible for the possible long term follow up of the offspring during youth, adolescence and adulthood.

Tone Shetelig Løvvik, Sven M. Carlsen and Eszter Vanky will have equal access and possibility to use the data and biological material collected in the possible follow-up study/s of the offspring.

We will create a consortium study group consisting of the principal investigator from each participating country and Sven M. Carlsen and Eszter Vanky. The major focus of this consortium group should be to get the highest scientific value possible out of the biological material gathered during the PregMet 2 study.

If any of the investigators have an idea for a project, she/he can present a protocol and apply the consortium group for material from the study. Given the protocol has received an

ethical approval and there is no conflicting interest with the planned study and sub-studies the consortium group should as a general rule approve the study if it is sound and of superior scientific value. However, the consortium study group should always balance approval for projects against the fact that the long term interest for the biological material sampled in the PregMet 2 study will increase with time due to the uniqueness of the material and the possibility to follow up the mothers and children.

Ethics

Our calculations on the effect of metformin on late miscarriages and preterm deliveries based on a conservative estimate from former studies, indicates a potentially cheap, safe and effective prevention of preterm deliveries in PCOS women. Metformin is cheap and the patent protection period expired many years ago. This means that the pharmaceutical industry does not find it worthwhile conducting studies on possible metformin effects in pregnancy.

Based on data the NNT to prevent one preterm birth is around 10. However, based on our conservative power calculation the NNT is around 20 to prevent a preterm birth. If a positive effect of metformin on preterm deliveries can be proven in a large-scale multicentre study, it would be the most cost effective preventive treatment ever. Given, the short and long term benefit for the patients and the families involved and the potentially large economic savings for the society, it seems unethical not to perform the study.

Metformin has never been reported to be toxic or do any harm to the fetus or to the mother. This was also confirmed in the PregMet 1 study. Metformin do however have some gastrointestinal side effects that in some cases can be unpleasant.

In Norway or other Nordic countries, there is no specific follow up program for PCOS women in pregnancy. Given the extensive and structured follow up during pregnancy described in the present study, of a group of women with increased risk of pregnancy complications and suboptimal pregnancy outcome, we believe that participating in the PregMet 2 study will be beneficial also for the women and fetuses allocated to the placebo group.

Future plans

It is our intention to follow up both the women and their children. This includes:

- Follow up the participants with a questionnaire 1 year after delivery about their general health and breast feeding experiences
- To follow up the mothers long term to study the impact of GDM on long term glucose homeostasis.
- To follow up the offspring in early childhood and adolescence regarding endocrine and metabolic status.
- To merge data base, such as the Medical Birth Registry with data from the PregMet 2 study.
- Perform genetic analyses on whole blood and placenta,
- Perform additional analyses on inflammatory markers and hormones.

For the follow up studies separate protocols and applications for regulatory authorities will be prepared.

Reference List

Reference List

1. Vanky E, Salvesen KA, Heimstad R, Fougner KJ, Romundstad P, Carlsen SM. Metformin reduces pregnancy complications without affecting androgen levels in pregnant polycystic ovary syndrome women: results of a randomized study. Hum Reprod 2004;19:1734-40.

2. Vanky E, Stridsklev S, Heimstad R, Romundstad P, Skogoy K, Kleggetveit O, et al. Metformin Versus Placebo from First Trimester to Delivery in Polycystic Ovary Syndrome: A Randomized, Controlled Multicenter Study. J Clin Endocrinol Metab 610-2010;95:E448-55.

3. Asuncion M, Calvo RM, San Millan JL, Sancho J, Avila S, Escobar-Morreale HF. A prospective study of the prevalence of the polycystic ovary syndrome in unselected Caucasian women from Spain. J Clin Endocrinol Metab 2000;85:2434-8.

4. Franks S. Polycystic ovary syndrome. N Engl J Med 28-9-1995;333:853-61.

5. Knochenhauer ES, Key TJ, Kahsar-Miller M, Waggoner W, Boots LR, Azziz R. Prevalence of the polycystic ovary syndrome in unselected black and white women of the southeastern United States: a prospective study. J Clin Endocrinol Metab 1998;83:3078-82.

6. Consensus. Revised 2003 consensus on diagnostic criteria and long-term health risks related to polycystic ovary syndrome (PCOS). Hum Reprod 2004;19:41-7.

7. Boomsma CM, Eijkemans MJ, Hughes EG, Visser GH, Fauser BC, Macklon NS. A meta-analysis of pregnancy outcomes in women with polycystic ovary syndrome. Hum Reprod Update 2006;12:673-83.

8. Bjercke S, Dale PO, Tanbo T, Storeng R, Ertzeid G, Abyholm T. Impact of insulin resistance on pregnancy complications and outcome in women with polycystic ovary syndrome. Gynecol Obstet Invest 2002;54:94-8.

9. de Vries MJ, Dekker GA, Schoemaker J. Higher risk of preeclampsia in the polycystic ovary syndrome. A case control study. Eur J Obstet Gynecol Reprod Biol 1998;76:91-5.

10. Gjonnaess H. The course and outcome of pregnancy after ovarian electrocautery in women with polycystic ovarian syndrome: the influence of body-weight. Br J Obstet Gynaecol 1989;96:714-9.

11. Glueck CJ, Goldenberg N, Wang P, Loftspring M, Sherman A. Metformin during pregnancy reduces insulin, insulin resistance, insulin secretion, weight, testosterone and

development of gestational diabetes: prospective longitudinal assessment of women with polycystic ovary syndrome from preconception throughout pregnancy. Hum Reprod 2004;19:510-21.

12. Carlsen SM, Romundstad P, Jacobsen G. Early second trimester hyperandrogenemia and subsequent preeclampsia: A prospective study. Acta Obstet Gynecol Scand 2004;84:117-21.

13. Serin IS, Kula M, Basbug M, Unluhizarci K, Gucer S, Tayyar M. Androgen levels of preeclamptic patients in the third trimester of pregnancy and six weeks after delivery. Acta Obstet Gynecol Scand 2001;80:1009-13.

14. Steier JA, Ulstein M, Myking OL. Human chorionic gonadotropin and testosterone in normal and preeclamptic pregnancies in relation to fetal sex. Obstet Gynecol 2002;100:552-6.

15. Tang T, Lord JM, Norman RJ, Yasmin E, Balen AH. Insulin-sensitising drugs (metformin, rosiglitazone, pioglitazone, D-chiro-inositol) for women with polycystic ovary syndrome, oligo amenorrhoea and subfertility. Cochrane Database Syst Rev 2009;CD003053.

16. Glueck CJ, Phillips H, Cameron D, Sieve-Smith L, Wang P. Continuing metformin throughout pregnancy in women with polycystic ovary syndrome appears to safely reduce first-trimester spontaneous abortion: a pilot study. Fertil Steril 2001;75:46-52.

17. Glueck CJ, Wang P, Goldenberg N, Sieve-Smith L. Pregnancy outcomes among women with polycystic ovary syndrome treated with metformin. Hum Reprod 2002;17:2858-64.

18. Glueck CJ, Wang P, Kobayashi S, Phillips H, Sieve-Smith L. Metformin therapy throughout pregnancy reduces the development of gestational diabetes in women with polycystic ovary syndrome. Fertil Steril 2002;77:520-5.

19. Glueck CJ, Pranikoff J, Aregawi D, Wang P. Prevention of gestational diabetes by metformin plus diet in patients with polycystic ovary syndrome. Fertil Steril 2008;89:625-34.

20. Jakubowicz DJ, Iuorno MJ, Jakubowicz S, Roberts KA, Nestler JE. Effects of metformin on early pregnancy loss in the polycystic ovary syndrome. J Clin Endocrinol Metab 2002;87:524-9.

21. Nawaz FH, Khalid R, Naru T, Rizvi J. Does continuous use of metformin throughout pregnancy improve pregnancy outcomes in women with polycystic ovarian syndrome? J Obstet Gynaecol Res 2008;34:832-7.

22. Legro RS. Metformin during pregnancy in polycystic ovary syndrome: another vitamin bites the dust. J Clin Endocrinol Metab 2010;95:5199-202.

23. Hack M, Fanaroff AA. Outcomes of children of extremely low birthweight and gestational age in the 1990's. Early Hum Dev 1999;53:193-218.

24. Tucker JM, Goldenberg RL, Davis RO, Copper RL, Winkler CL, Hauth JC. Etiologies of preterm birth in an indigent population: is prevention a logical expectation? Obstet Gynecol 1991;77:343-7.

25. Goldenberg RL, Culhane JF, Iams JD, Romero R. Epidemiology and causes of preterm birth. Lancet 5-1-2008;371:75-84.

26. Olsen P, Laara E, Rantakallio P, Jarvelin MR, Sarpola A, Hartikainen AL. Epidemiology of preterm delivery in two birth cohorts with an interval of 20 years. Am J Epidemiol 1-12-1995;142:1184-93.

27. Bryce J, Boschi-Pinto C, Shibuya K, Black RE. WHO estimates of the causes of death in children. Lancet 26-3-2005;365:1147-52.

28. Morken NH, Vogel I, Kallen K, Skjaerven R, Langhoff-Roos J, Kesmodel US, et al. Reference population for international comparisons and time trend surveillance of preterm delivery proportions in three countries. BMC Womens Health 2008;8:16.

29. McCormick MC. The contribution of low birth weight to infant mortality and childhood morbidity. N Engl J Med 10-1-1985;312:82-90.

30. Petrou S. Economic consequences of preterm birth and low birthweight. BJOG 2003;110 Suppl 20:17-23.

31. Eilertsen TB, Vanky E, Carlsen SM. Increased prevalence of diabetes and polycystic ovary syndrome in women with a history of preterm birth: a case-control study. BJOG 2012;119:266-75.

A. **Report of a WHO expert committee.** “The prevention of perinatal mortality and morbidity.” WHO Technical Report Series; 1970; 457: 1- 660

B. **Fødsler i Norge gjennom 30 år** (1967-1996). Bergen: Medisinsk fødselsregister 1997

C Centers for disease Control and prevention (CDC). Preterm singleton births; United States 1989-1996. Morbidity and Mortality weekly reports. 1999; Mar 12; 48 (9): 185-9

D Morken NH, Introduction to the doctoral Thesis:, Public Health Aspects of Preterm Birth 2008 (ref 20-22 I intro)

E Morken NH, Introduction to the doctoral ThesisPublicMorken, Public Health Aspects of Preterm Birth 2008 (ref 16 og 40-50 I intro)

F Figures from the Economy section of the Dept. of Pediatrics, St.Olavs Hospital

Appendix I

Blood Collection Procedure, version II:

Vein puncture

Fasting: 10-16 h fasting

Procedure: The median cubital and cephalic veins of the arm are used most frequently for blood samples.

- In 10 containers: VACUETTE SERUM Gel Red top /yellow ring 3 ml (or 6 - 7 containers à 5 ml) ○ In 1 container: BD Vacutainer Plus EDTA 10 ml (this is for the "buffy coat") ○ In 1 container VACUETTE Li-Heparin PLASMA Gel green top/Yellow ring – Glucose to be analyzed at the local hospital.
- Results to be recorded in the Web CRF

Centrifuge and storage:

Serum

- The ten serum containers (red top/ yellow ring) are to be turned upside down a couple of times.
- Leave them in room temperature for approximately 20 minutes (max 2 hours) until the blood is completely clotted.
- The centrifuge at 1800 G in 10 minutes (or at 2800G for 5 minutes)
- Centrifuged serum is not to be left in room temperature for more than max two hours!!
- In a fridge it can be left no more than 24 hours.
- Serum is pipetted into cryotubes = NUNC tubes (1, 8 ml) ***(8-10 vacuette serum gel containers fill approximately 9-10 cryotubes.)***
- The cryotubes with serum ought to be frozen as soon as possible.

Plasma and Buffycoat (cells)

- The EDTA container (Purple top/black ring) are to be turned up-side-down 8-10 times and centrifuged (within 6 hours) at 1800 G in 10 min.
- Plasma is pipetted into 3 cryotubes (obs: only plasma, no cells) and buffycoat is then gently pipetted into 1 tube, mixed and split into 2 cryotubes.

- ***The cryotubes with plasma and buffycoat ought to be frozen as soon as possible.***

All Cryotubes

- Each cryotube tube should be marked with: a barcode given from The Regional Biobank of Mid-Norway, henceforth called the Biobank. The cryotubes will be marked by different colored insert cryocolor coders, one color for each visit, to ease the analyzing process after finishing the study.

- The cryotubes tubes are to be stored in appropriate stands or boxes of 10 x 10 tubes (One box per patient). Preferably at – 80 °C If no access to – 80 °C freezer then use - 20°C (minimum)
- The cryotubes tubes are stored at the study site until the patient is through the study. (Until she has delivered).
- The boxes will then be shipped by World Courier to Tone Shetelig Løvvik/ or study midwife, Dept. of Obstetrics, St. Olavs Hospital, 7006 Trondheim, Norway (phone
+47 72575715, E-mail pregmet2@stolav.no) on dry ice. The package will be received and put directly into -80 °C freezer. Shipment will be ordered directly by the investigator at the actual study site on The World Courier Web-site(http://www.worldcourier.com/)

At each visit ; the aim is to collect

10 cryotubes (á 1,8 ml) with serum

2 cryotubes (á 1,8 ml) with buffy coat

3 cryotubes (á 1,8 ml) with plasma

This comes from one 10 ml EDTA container

Color codes

- Insert Cryocolour coder:
  approximately 14-15 tubes/visit
  ○ Inclusion: red ○ Week 19: yellow ○ Week 28: blue ○ Week 32: green ○ Week 36: brown
  ○ Umbilical cord blood + maternal vein at delivery: purple (Trondheim, Uppsala, SÖS)

Appendix II, 2-hours glucose tolerance test

Procedure

2-hours glucose tolerance test:

Precautions:

- Fasting: 10-16 hours. It is most convenient that the patient eats regular supper the night before, and meet for the glucose tolerance test the morning after
- She is allowed to drink water
- Any regular medication is to be taken as usual with a little water.
- The patient should sit in a comfortable chair during the test (2 hours).
- She should not smoke before the test.

Procedure:

- There shall be taken venous blood sample (from fossa cubiti) for glucose. NB! Not capillary blood!
- Then the patient drinks 75 grams of glucose dissolved in 300 ml of water. Often preferred added ice cubes and lemon juice!
- A new venous blood sample taken after 2 hours for determination of glucose.
- The analysis must be done on plasma or serum. Not of whole blood! This must be clarified with the laboratory.
- The sample should be analyzed immediately (within a few hours).

Sample Containers:

- VACUETTE Plasma Gel - green cap / yellow ring or equivalent

Results are put into CRF

The glucose 2h tolerance test is not performed if the patient has been diagnosed with gestational diabetes in the current pregnancy

Appendix III Ferriman-Gallwey score

Appendix IV Norwegian labels for study medicine

ETIKETTER TIL METFORMIN / PLACEBO

TIL KLINISK UTPRØVNING
Metformin 500 mg / Placebo
100 tabletter
Weifa / Sykehusapoteket i Trondheim ,St. Olavs Hospital
Utprøverinitiert studie: Dr. Eszter Vanky, St. Olavs Hospital , Trondheim
Telefon: 73 87 38 19

1 tablett 2 ganger daglig i 7 dager, 2
tablett 2 ganger daglig inntil fødsel
Svelges hele med vann.
Tas helst til måltid.

Studiekode: The PregMet 2 Study
Randomiseringsnr:______________

Batchnr.:___________

Ansvarlig lege:______________

OPPBEVARES UTILGJENGENLIG FOR BARN
Holdbarhetsdato:_________________
Pakket av Sykehusapoteket i Trondheim, St Olavs Hospital
