# Supplementary material for: Metformin exposure, maternal PCOS status and fetal venous liver circulation: A randomized, placebo-controlled study
Source: PLoS One. 2022 Jan 28;17(1):e0262987. doi: 10.1371/journal.pone.0262987 (PMC8797196; doi:10.1371/journal.pone.0262987)
Supplement: Attachment — Submitted filename: Response to Reviews.docx [file pone.0262987.s005.docx]

Dear Stephen L Atkin

Thank you for the opportunity to improve our manuscript. We highly appreciate the meticulous work of the reviewers and have the following comments:

Review Comments to the Author

Reviewer #1: The manuscript entitled ‘Metformin exposure, maternal PCOS status and fetal venous liver circulation: a randomized, placebo-controlled study’ with the aims to compare 75 fetal venous liver blood flow in both metformin versus placebo-exposed fetuses of mothers with PCOS, and all fetuses of PCOS mothers to a low-risk reference population.

This is quite an interesting study, however, the manuscript presentation can be further improved.

Comments
Material and Methods
Line 129 and 161, Table 1 and Table 2 to be placed in the results section.

***Response:*** *We chose to place these tables in the M&M section due to the nature of their content, as well as that is the place where they were first referred to. These tables show the baseline of the women included in the current study, as well as the reference group, in addition to maternal and fetal outcomes. Moving this information to the result section could indicate that the reference group was also recruited in parallel to this study, which was not the case. Also, PLOS-one manuscript formatting guidelines state that “Tables should be included directly after the paragraph in which they are first cited.”*

*We do not mind moving these tables to the result section, but since the PLOS guidelines statement regarding tables are as such, we ask the editor for advice on where these tables should be placed.*

Statistical analysis
Line 208, proper citation for statistical software including publisher name to be included.

***Response: The*** *proper citation has been done according to publisher (https://www.ibm.com/support/pages/how-cite-ibm-spss-statistics-or-earlier-versions-spss)*

Ensure all statistical tests mentioned in the statistical analysis section are clearly denoted in the results section table(s). 1 or 2-tailed test to be stated.

***Response: The*** *2-tailed p-values have been stated in the M&M and relevant tests have been denoted in all tables.*

Results
Line 228-230, for the crude and adjusted analysis, the tables to be clearly indicated. The adjusted variables maternal blood pressure, BMI, and gestational weight gain to be denoted in the table footnote.

***Response:*** *We present the crude/unadjusted table since no changes were found after adjustment for these factors. This is now clarified in the table (table 3) and in the manuscript.*

*We further decided to remove gestational weight gain from the adjustment since this was more likely to be an outcome of metformin treatment and not a baseline parameter. Since BMI at inclusion was border significant only, we ran the adjusted analyzes, however it had no impact on the results.*

Information on whether multicollinearity was assessed between the covariates to be stated.

***Response: We performed appropriate statistical*** *analyses and found multicollinearity for systolic and diastolic blood pressure. We therefore adjusted for each of them separately as well as combined without finding any influence on the results (overlapping confidence intervals). We therefore chose not to present the adjusted numbers.*

Line 128 Table 1, for some p values, technically p value cannot be zero (to use symbol p<) Symbol ¶ to be denoted in the table. Since the p value is written on the top of the table, individual ‘p=’ to be omitted. Likewise with Table 3.

***Response:*** *The duplications have been removed (p=) and correct values have been inserted (p<). Symbol ¶ has been denoted in table 1.*

Line 161 Table 2, the decimal points for the p value to be standardized.

***Response:*** *Three decimals have been standardized in all analyzes*

Nonetheless, based on CONSORT statement, all statistical tests for baseline group comparison to be avoided (Table 1 & Table 2).

***Response:*** *Since this study was a substudy of a larger RCT, we decided to run these analyses between the groups since the differences found do have a physiological potential to affect the fetal liver blood flow. These differences in baseline were not found in the main study thus indicating a slightly skewed inclusion. We therefore ask not to omit the statistical comparison.*

Table 1 & 2, at least 1 decimal point for percentage figures.

***Response:*** *Adjustments have been performed accordingly.*

Line 243 Table 3, what the figures in bracket represents to be clearly denoted. More information on the GLM to be clearly highlighted/denoted e.g dependent variable(s), factors/covariates, multiple comparison correction (if any) etc, Adjusted variables to be indicated in the table footnote and 95%CI to be included for the p values.

***Response:*** *We have clarified the content of the brackets.*

*We used GLM using each flow measurement (z-score) as a dependent variable, randomization as fixed factor and BMI, systolic and diastolic blood pressure as covariates, first individually, then combined. This is further elaborated in the M&M section.*

Line 253 Table 4, mean and sd to be clearly denoted.

***Response:*** *The denotation has been revised accordingly.*

Figure 1 requires improvement by incorporating details of study design i.e. number of groups in the study.

***Response:*** *An updated consort diagram has been incorporated in the manuscript clarifying the randomization groups.*

Reviewer #2: Please use the space provided to explain your answers to the questions above. You may also include additional comments for the author, including concerns about dual publication, research ethics, or publication ethics. (Please upload your review as an attachment if it exceeds 20,000 characters) (Limit 200 to 20000 Characters)

please clarify the number of patients enrolled to this particular study in the abstract and put to the abstract that this paper is a part of larger study

***Response:*** *This request has been attended to and the text is revised accordingly.*
