# Supplementary material for: Metformin exposure, maternal PCOS status and fetal venous liver circulation: A randomized, placebo-controlled study
Source: PLoS One. 2022 Jan 28;17(1):e0262987. doi: 10.1371/journal.pone.0262987 (PMC8797196; doi:10.1371/journal.pone.0262987)
Supplement: Attachment — Submitted filename: Response to Reviewers 2nd review.docx [file pone.0262987.s006.docx]

Dear Stephen L Atkin

Thank you for the important feedback we have received, especially from the reviewers, on our last revision. We have attended the last comments and submit now the latest version.

Sincerely

On behalf of the authors

Sindre Grindheim

Review Comments to the Author

Reviewer #1: Minor comments
Table 1 Parity & GDM, decimal points for percentage figures.

*Comment: This has been adjusted accordingly.*

It is best to present the findings in adjusted form even though it has no impact on the results.

*Comment: The adjusted results are now presented in the table and adjusted in the text.*
